# The Supramolecular Buildup of Eumelanin: Structures, Mechanisms, Controllability

**DOI:** 10.3390/ijms18091901

**Published:** 2017-09-06

**Authors:** Anne Büngeler, Benjamin Hämisch, Oliver I. Strube

**Affiliations:** 1 Biobased and Bioinspired Materials, Department of Chemistry, Paderborn University, 33098 Paderborn, Germany; anneb2@mail.upb.de; 2 Department of Physical Chemistry, Paderborn University, 33098 Paderborn, Germany; haemisch@mail.uni-paderborn.de

**Keywords:** melanin, enzymes, supramolecular structure, nanoparticles, autodeposition

## Abstract

Research on the supramolecular buildup of eumelanin has gained high momentum in the last years. Several new aspects regarding the involved structures and mechanisms have been established, which has led to a better understanding of the entire process. This review intends to provide a clearly laid-out summary of previous and new findings regarding structures, mechanisms, and controllability. With respect to materials applications, the aspect of controllability is of supreme importance. A focus of this review is therefore set on a novel method with high potential for specific synthesis of various, isolated particle morphologies. Finally, open questions and possibilities for their elucidation are discussed.

## 1. Introduction

Melanins, especially the most common variant, eumelanin, are a highly relevant class of biomacromolecules. Besides their importance in medical aspects as a key pigment of biological organisms [1,2], they also exhibit a large number of unique properties with applications in physics and material sciences [3,4,5,6]. The most important ones are free radical scavenging [7], paramagnetism [8,9], broad band absorption and very low fluorescence [10], drug delivery capability [11,12,13], and hybrid electrical conductivity [14,15]. This immensely high potential originates from the chemical versatility on the molecular level as well as from the distinct supramolecular structure of the resulting particles.

**Figure 1:**
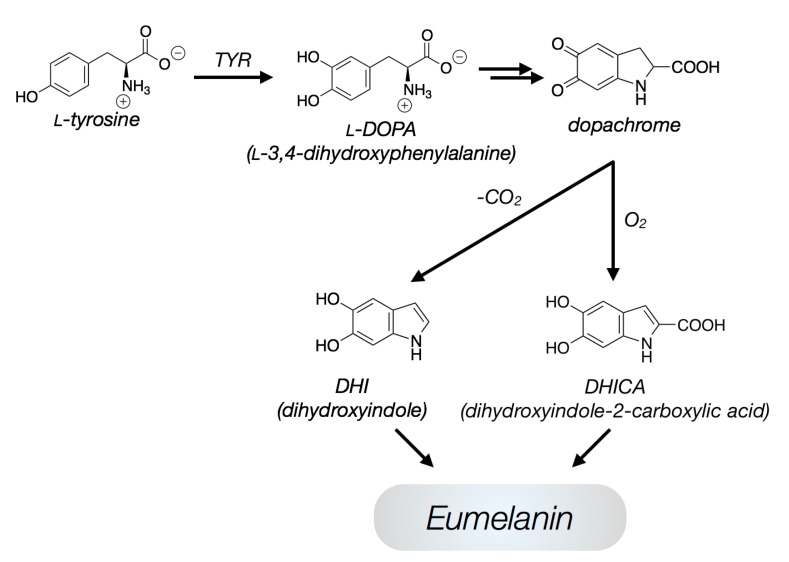
Eumelanin synthetic pathway; simplified version.

In vivo, eumelanin is formed from l-tyrosine with the involvement of various enzymes. The most important enzyme is tyrosinase (TYR), an oxidase which, in the absence of other enzymes (tyrosinase related proteins: Tyrp-1, Tyrp-2), can catalyze the entire reaction cascade with the significant intermediates l-3,4-dihydroxyphenylalanine (l-DOPA) anddopachrome. The final eumelanin oligomer consists of various types of monomers which exhibits different oxidation states, derived from 5,6-dihydroxyindole (DHI). The complete mechanism is described in the literature [16] and a simplified version is shown in Figure 1.

In the last decades, a lot of research has been conducted to decode the binding sites of the monomers, the composition of the comonomers, and the degree of polymerization. The current state is that eumelanin forms oligomeric structures with chain lengths of up to a few tenth of monomers and variable chemical composition [17,18,19]. Additionally, the kinetics of the reaction cascade has also attracted high attention. For example, it was revealed that the pH and concentration of copper ions significantly retard or accelerate the final cascade step from dopachrome to DHI or 5,6-dihydroxyindole-2-carboxylic acid (DHICA) [20].

Another important aspect that has been investigated lately is the control of DHI or DHICA units in the oligomers. It was found that in nature the Tyrp-2 enzyme is the main tool, but also copper and zinc ions influence the DHICA concentration in DOPA-melanins [21,22]. In this context, it was also found that both monomers are responsible for different material properties, allowing fine-tuning of those in synthetic approaches. The antioxidant and paramagnetic properties of eumelanin pigments are strongly affected by the ratio of DHI to DHICA. Pigments with a higher DHICA content showed a decreased visible light absorption and paramagnetic response but on the other hand enhanced antioxidant properties. The reason for these differences were attributed to peculiar features of carboxylated units. Due to the presence of an additional carboxy-unit, the number of reactive sites for polymerization is lowered based on sterical properties. Furthermore, the additional negative charge alters the structural properties of oligomers and their applicability for post-synthetic modifications. Finally, a lower oxidation potential is observed [23].

While the fundamental aspects on the molecular structure has been well elucidated, the supramolecular buildup from the oligomeric units to the observed melanin particles is far less understood and remains a challenging task. However, exact knowledge in this field is of great importance for the applicability of eumelanin in functional materials, as incisively punctuated by d’Ischia et al. Breaking this aggregation without affecting the primary unit structure or properties is the secret which unlocks the potential of these materials [4]. Thus, there has been an increasing interest on this aspect in the last years, and some significant aspects could already be elucidated. This review intends to give a roundup on revealed structures, mechanisms, and possible control features as well as open questions on the supramolecular buildup of eumelanin.

## 2. Structures

### 2.1. General Remarks

Natural eumelanin from human skin, hair, and eyes has been examined by various microscopy methods. These have shown that it consists of particles with a very uniform size of about 200 nm in diameter. Studies on sepia melanin from *S. officinales* showed the same spherical structure and identical sizes [24,25,26]. Furthermore, a substructure of approximately 20 nm could be discovered in studies of melanosomes isolated from bovine RPE cells [25]. This was confirmed as a very similar particle-like substructure was observed within the spherical sepia melanin granules by means of atomic force microscopy [27]. Those results led to the assumption that the naturally occurring 200 nm spherical structure is built up by an aggregation of a smaller particle species.

Fibril-like structures from sepia eumelanin could be generated by drying a low concentrated dispersion on mica. The structures could not be returned to the natural morphology after removal from the mica surface. This behavior of the hydrophobic eumelanin on a hydrophilic surface also underlined that the eumelanin structure is not a large cross-linked polymer, but rather a composite of aggregated oligomer building blocks [25,28].

Synthetic melanins can be made by a wide variety of techniques [29]. The most common ones are the auto oxidative approach at increased pH, oxidation with ferrocyanide, and the biomimetic synthesis with tyrosinase. Additionally, nearly all stages of the reaction cascade have been used as starting precursor, the most prevalent ones are l-tyrosine, l-DOPA, DHI, and DHICA. Use of the latter two, however, limits the chemical variety of the oligomers and changes the material properties in relation to natural eumelanin.

The biomimetic synthesis of eumelanin with tyrosinase is the method most equal to the in vivo process. Thus, the resulting product shows the highest similarity to the natural eumelanin in both molecular and supramolecular structure. It mostly leads to spherical particles with diameters of about 200 nm, whose appearance is similar to that of sepia eumelanin [28,30,31].

The resulting supramolecular structure of other synthetic eumelanins is strongly dependent on the choice of reaction conditions, especially pH and presence or absence of enzyme. The morphology may be similar to the natural pendent, but can also be completely different, e.g., in the form of fibrils or as an amorphous solid [7,25,32]. Particularly, the auto oxidative process mostly results in no defined particle structure. There has also been a report of a disc-like structure of DHI-melanin with thicknesses around 55 nm. However, their lateral dimension and visual confirmation e.g., by microscopy techniques, remain open questions [33].

It has also been shown by Lampel et al. that a tyrosine-containing tripeptide might also lead to melanin-like materials in certain combinations [34,35]. Interestingly, the position of tyrosine in the peptide seems to determine the supramolecular structure via self-assembly on the micrometer scale, where films or fibrils are gained. The nanostructure of the species, similar to other melanins, however, was not considered. This might be a very interesting aspect for future examinations.

There have been several reports of melanin-like materials with different particle morphologies and sizes [36]. Especially, the use of dopamine as precursor is highly popular due to the easy synthesis and characterization. Moreover, the structural features of dopamine-melanin are controllable to a certain amount. In this manner, it was possible to gain spherical dopamine-melanin particles with variable diameters [10,37,38], thin polymeric films, and highly adhesive coatings for surface modifications [39,40,41]. Although these approaches usually take reference to eumelanin, polydopamine differs structurally from natural or biomimetic eumelanin, since it does not consist of the natural abundance of monomers. For this reason, the properties are not comparable with those of real eumelanin.

**Figure 2:**
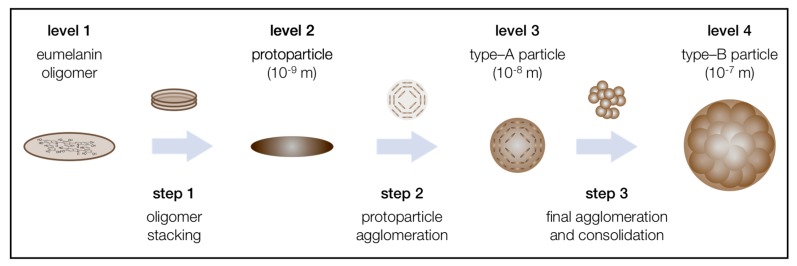
Most recent version of the three-step, four-level hierarchical buildup mechanism of natural and biomimetic eumelanin, based on the state of literature [31].

For natural and biomimetic eumelanin, which shall be the main focus in the following, a hierarchical aggregation mechanism has already been proposed in the mid-1990s, although several aspects were not confirmed experimentally [10]. Recently, the examination of the supramolecular buildup of biomimetic eumelanin has gained new momentum. In this context, all three particle types, proposed in the hierarchical four level buildup, could be synthesized in as nonaggregated entities [31,42,43]. This enabled the proposal of an updated version for the hierarchical buildup, consisting of fours levels and three steps (Figure 2). Each aggregation step increases the particle size by one order of magnitude: The oligomeric sheets form protoparticles (10^−9^ m), which then arrange in an onion-like structure and densify into the smaller, spherical type-A particles (10^−8^ m), and finally undergo aggregation to the larger, again spherical type-B particles (10^−7^ m). In the following, the levels and aggregation steps are discussed in detail.

### 2.2. Level 1—Oligomeric Sheets

The oligomeric sheets define the end of the reaction cascade and pose as the starting material for the supramolecular buildup. As mentioned above, they show high chemical diversity, though the monomers are all derived from DHI/DHICA. The linkage between the monomers can occur at various positions as well [18,19]. Until recently, it was believed that the degree of polymerization falls in the range of 6–8 monomeric units. The latest MALDI-TOF MS (matrix-assisted laser desorption/ionization-time of flight mass spectrometry) investigations, though, have shown that in fact several tens of monomers may be combined in one entity [17].

It was also found lately that the amount of DHI and DHICA within the oligomers plays a crucial role in the first aggregation step and influences the geometry of the stacks. While DHI-rich oligomers preferably undergo the long proposed π-stacking, DHICA-rich oligomers were found to undergo random bundling due to their non-planar structure [23].

### 2.3. Level 2—Protoparticles

The first particle structure in the hierarchical buildup is often referred to as protoparticles or protomolecules. As it has since been shown that the structures are in fact particles [43], the term protoparticle seems more appropriate, whereas protomolecule might very well be an intermediate structure, formed during the stacking of oligomers.

The existence of protoparticles was first revealed in the mid of 1990s by Eisner et al. via X-ray diffraction measurements of amorphous melanin. It was found that the oligomeric sheets undergo π-stacking, which forms the protoparticle. Theoretical predictions estimated the size of those protoparticles to be about 2 nm [44].

The first visual confirmation of protoparticles was delivered by Watt et al. via transmission electron microscopy (TEM). It was shown that the protoparticles can still be distinguished within the final eumelanin particles as an individual structure. It was observed that they undergo a circular, onion-like arrangement. The size of the protoparticles, however, was found to be around 5 nm and therefore slightly larger than predicted [27]. A similar structure was observed for dopamine-melanin by Chen et al. again via TEM investigation, which was also supported by theoretical determinations [45]. This might be another proof for the kinship of dopamine-melanin to other melanins.

In the past few years, numerous studies via atomic force microscopy [25,28], X-ray diffraction [46], or mass spectroscopy [17,47] were performed to investigate the eumelanin structure. Most of them support the proposed stacking model of Eisner et al., but the existence of protoparticles as a distinct level could not be shown again for natural or biomimetic eumelanin.

The first selective synthesis of protoparticles as individual entities was achieved by Strube et al. via the method of enzyme mediated autodeposition, which will be explained in detail in Chapter 4. It could be verified that the protoparticle is an independent particle species with a rod-like structure and a length of about 6 nm [43].

### 2.4. Level 3—Type-A Particles

The particles that define the third level (referred to as type-A particle in the following), are probably generated via an onion-shell-like arrangement of a plurality of protoparticles [27]. Originally, their existence as a distinct structure could only be shown indirectly via atomic force microscopy (AFM), transmission electron microscopy (TEM), or scanning electron microscopy (SEM), mostly on sepia melanin particles. They were observed as a substructure of the common 200 nm spheres, but never as an isolated intermediate state. Their size was estimated to be several tens of nanometers [25,27].

Particle size of dopamine-melanins can be tailored in the range of several tens of nm up to around 400 nm [37,45,48]. Thus, particles in the shape and size of type-A structures can be gained. However, it is unclear to what amount the buildup process is comparable to that of natural eumelanin. Via a specific drying method of DHI-melanin, d’Ischia et al. obtained particle-like structures with sizes fitting to that of type-A particles. However, the particles showed partial aggregation [7].

The first direct proof of a distinct particle structure was again delivered via enzyme mediated autodeposition (see chapter 4) of eumelanin. Here, completely individual, biomimetic type-A particles could be prepared in various amounts. This deposition was limited to a support surface, but could be used to verify their existence as a distinct intermediate step [42]. The size of the spherical type-A particles could also be defined and lies in the range of 40 ± 10 nm. The protoparticles as a substructure could not be observed, as TEM measurements, which could confirm the previously described onion-like structure, are still pending. The size and the shape of type-A particles could be confirmed via light scattering measurements [31].

### 2.5. Level 4—Type-B Particles

The last level in the supramolecular buildup of eumelanin is the most commonly occurring and investigated species which is called type-B particle in the following. They are spherical structures and show a very uniform size of about 200 nm in diameter [27,28,30].

Electron microscopy investigations sometimes show a clearly visible substructure of type-A particles, which confirms the direct aggregation as building mechanism of type-B particles. The distinctiveness of this substructure varies from loosely aggregated type-A particles to fully smooth type-B spheres [25,31]. The reasons for the varying degree of consolidation is not yet fully understood. However, a combination of time-resolved light scattering and scanning electron microscopy has shown that the consolidation of the particles is much slower than their initial aggregation buildup [31].

All structures beyond the size of 200 nm, which are sometimes mentioned in the literature, cannot be attributed to the concept or the definition of natural or synthetic eumelanin. Those are not pure eumelanin, but rather a combination of eumelanin with other biological substances, such as proteins [25], and are therefore defined as a natural composite material.

## 3. Mechanisms

### 3.1. General Remarks

The formation of the final eumelanin particle takes place over a three-stage agglomeration mechanism. Here, the individual particle levels are passed through until the process finally ends at the stage of type-B particles. This three-fold mechanism, presented in Figure 2, is based on π-stacking and subsequent, spontaneous aggregation [44]. Despite intensive research in the last decades, several mechanistic aspects are still unknown. In particular, the aggregation behavior and the formation of the individual species occurring uniformly in their size and shape are still not fully understood.

Complete understanding of the entire supramolecular buildup mechanism, however, is necessary to gain control over the synthesized structures. In this way, the specific synthesis of individual particle species could be achievable.

### 3.2. Agglomeration Step 1—From Level 1 to Level 2

In the first step, oligomeric sheets are assumed to undergo π-stacking and to form protoparticles. Theoretical predictions give the size of those graphene-like oligomeric stacks as 2 nm, consisting of three to four planar sheets. The spacing between the sheets accounts to roughly 0.3 nm, depending on the oxidation state of the monomers. In the fully reduced state, the spacing is lowered due to the enhanced formation of hydrogen bonds [44]. As the resulting sizes do not fit to that of observed protoparticles, those stacks most likely are only an intermediate species. For DHI-melanin, a three-step aggregation from oligomers towards protoparticles was proposed [49], that might lead to fitting magnitudes.

Another important aspect in the first step is the amount of DHI vs. DHICA within the oligomers. As mentioned before, both monomers promote a different agglomeration behavior [23]. DHI-rich entities promote the long described π-stacking due to their planar structure. DHICA entities on the other hand promote random agglomeration, which also increases water solubility of the melanin under certain conditions [23,33]. This interplay between different agglomeration mechanisms might, at least partially, explain the morphological differences between various melanin types, and the shape or size of protoparticles.

Despite those advances, many questions concerning the formation of the protoparticles are still unresolved. The most important questions in this case are whether the generation of protoparticles is an aggregation or agglomeration process, and if this buildup step is reversible. In addition, it is important to gain a kinetic understanding of the first particle buildup reaction step in order to develop the possibility to stop the process at this stage and thus to generate the protoparticles in an isolated stable form, besides the immediate deposition via enzyme mediated autodeposition.

### 3.3. Agglomeration Step 2—From Level 2 to Level 3

In the next step, a plurality of protoparticles generates the compact and spherical type-A particles, via the previously described onion-shell-like arrangement [27]. Until today, almost nothing is known about the mechanism of this buildup step. Clarification is desired but might pose particularly challenging, as the time for type-A buildup is probably in the range of seconds, as indicated by light scattering investigations [31]. Because the TEM investigations by both Watt et al. and Chen et al. showed that the protoparticles are still individually recognizable [27,45], it is particularly interesting to find out if this buildup can be reversed.

### 3.4. Agglomeration Step 3—From Level 3 to the Final Level 4

The final step in the supramolecular buildup of eumelanin particles is the formation of type-B particles. Via a combination of three different methods, namely scanning electron microscopy, time resolved light scattering, and enzyme mediated autodeposition, it was possible to identify the mechanism and the involved species [31]. The results confirmed the previously proposed aggregation of type-A particles as the driving force for this buildup step and exclude an ongoing growth of type-A particles. This might be a relevant difference to dopamine-melanin, where variable particle sizes and no substructures are observed.

**Figure 3:**
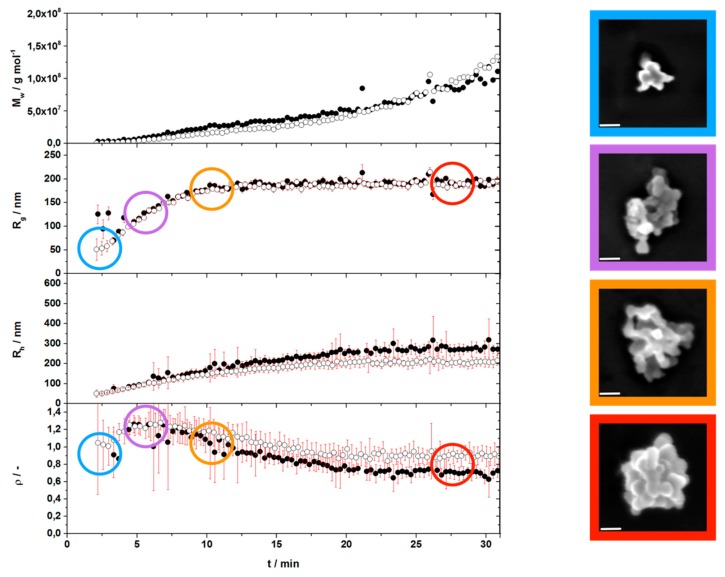
Two runs of each, evolution of molar mass, radius of gyration, hydrodynamic radius, and shape sensitive parameter over time; measured with time-resolved static and dynamic light scattering and the resulting scanning electron microscopy (SEM) pictures showing the ongoing aggregation with respect to reaction time. Each experiment was performed with fresh (●) and 24 h standing (○) tyrosinase solution. Scale bars of SEM pictures are 50 nm.

As shown in Figure 3, it became evident that the formation of an individual type-B particle takes only a few minutes of reaction time, while the exact kinetics depend on the precursor concentration. The entire process of particle buildup goes on, as long as precursor is available, which is shown by the ever-increasing value of *M*_w_ in the region of constant R_g_.

Additionally, the light scattering measurements gave information about the morphology of the growing particles. This can be gained from the shape sensitive parameter ρ, i.e., the ratio of R_g_ and R_h_. The ρ-parameter starts at 0.8–0.9, close to the value of compact spheres, and then passes a maximum at 1.2, beyond which it again decreases down to 0.7–0.9, eventually recovering the value of compact spheres after 15 min of reaction time as a constant plateau value [50,51].

**Figure 4:**
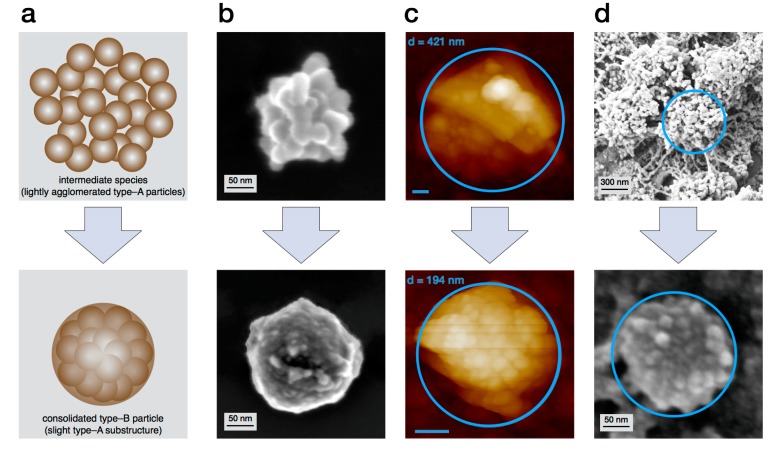
The final consolidation step during the buildup of type-B particles with (**a**) model pictures of lightly agglomerated type-A to consolidated type-B particles; (**b**) SEM picture of type-B eumelanin particles at different consolidation states; (**c**) atomic force microscopy (AFM) pictures of type-B particles in wet and dry condition state and (**d**) type-B particles generated via enzyme mediated autodeposition at different consolidation states [31]. Scale bars of AFM pictures are 50 nm.

Surprisingly, the light scattering determines the final particle radius to be around 200 nm i.e., a diameter of about 400 nm. This seeming discrepancy to the always observed diameters of 200 nm is explained with an additional consolidation step. Its existence was proven by AFM measurements in fluidic phase and enzyme mediated autodeposition (Figure 4). With both methods, the lightly agglomerated structure as well as the consolidated particle could be observed. Moreover, it was described that the process of consolidation might take up to 24 h [31].

## 4. Controllability

### 4.1. Motivation

The ability to selectively synthesize specific particle types of eumelanin in an isolated form is a highly-aspired aim of research about the supramolecular buildup. This is due to different particles sizes over three areas of magnification, and potential influences on material properties. This ability could enable numerous possibilities for application of this extraordinary biological material in physics and material science, especially in nanotechnology and life sciences.

### 4.2. Aggregation Breaking and Specific Syntheses in Solution

Natural eumelanin already exists in its final form (type-B particles) when it is isolated from its source [24,25,27]. Though, at the moment, there have been no earnest attempts to do so, one possible way to obtain lower level structures from this material is to break the aggregation. Future research might shine light on the possibility or impossibility to do so. The advantage would be to use the original biological material without any need for complicated syntheses.

In vitro eumelanin syntheses in solution, until now, have yielded either amorphous solids, highly coalesced particles, or type-B particles, similar to natural eumelanin [7,31,32]. The only way so far, that seems potentially able to achieve lower level structures is the biomimetic synthesis with tyrosinase. As the role of this enzyme is still not fully elucidated, its influence might very well have effects on the particle structures that can be exploited.

D’Ischia et al. investigated the influence of polyvinyl alcohol (PVA) during the buildup of different synthetic eumelanins [23,52]. It was shown that the presence of PVA increases water-solubility of eumelanin. This was attributed to an aggregation-inhibiting behavior of PVA, which is due to hydrophobic interactions between eumelanin particles and PVA molecules. Thereby polyvinyl alcohol acts as a stabilizing additive which encapsulates the growing eumelanin particles, and consequently prevents further particle aggregation, potentially enabling control over particle morphology.

A high variety of structures can also be obtained from dopamine-melanins. Although this class is much different from natural melanin in molecular structure as well as in properties, it is highly popular due to its easy feasibility. In recent years, a lot of research on dopamine-melanin has been performed to create eumelanin-like materials with specific physicochemical properties for applications like biosensing [53], drug delivery [54], light-harvesting systems [55], and surface modifications [40,41].

Another benefit of the dopamine approach is the possibility to address open questions regarding structure–property relationship in melanins, including correlations with natural eumelanin. A full understanding of the interaction between hierarchical structures and properties of melanin or melanin-like materials opens up the ability to control and design specific melanin materials with tailor-made properties for specific applications [49].

### 4.3. Specific Synthesis via Enzyme Mediated Autodeposition

**Figure 5:**
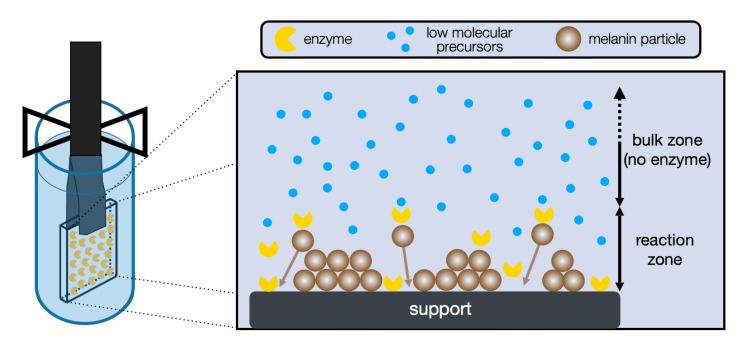
Concept of enzyme mediated autodeposition of eumelanin by immobilization via adsorption. Variation of reaction zone is achieved by choice of immobilization method and results in defined particle types. The reaction zone is indicated by the double arrow.

Recently, a novel approach for eumelanin synthesis has been developed with in situ formation and deposition of biomimetic emulation particles; the enzyme mediated autodeposition [42]. In this process, tyrosinase is tethered onto a support, which is subsequently immersed into a solution of a precursor (up to now, l-DOPA). The key concept of this method is to limit the tyrosinase mobility by immobilization. Thus, the entire enzymatic buildup of eumelanin occurs only in direct proximity to the support surface where the enzyme is present. The hydrophobic eumelanin particles deposit as soon as the buildup is complete due to hydrophobic interactions. Figure 5 shows the concept of this approach.

**Figure 6:**
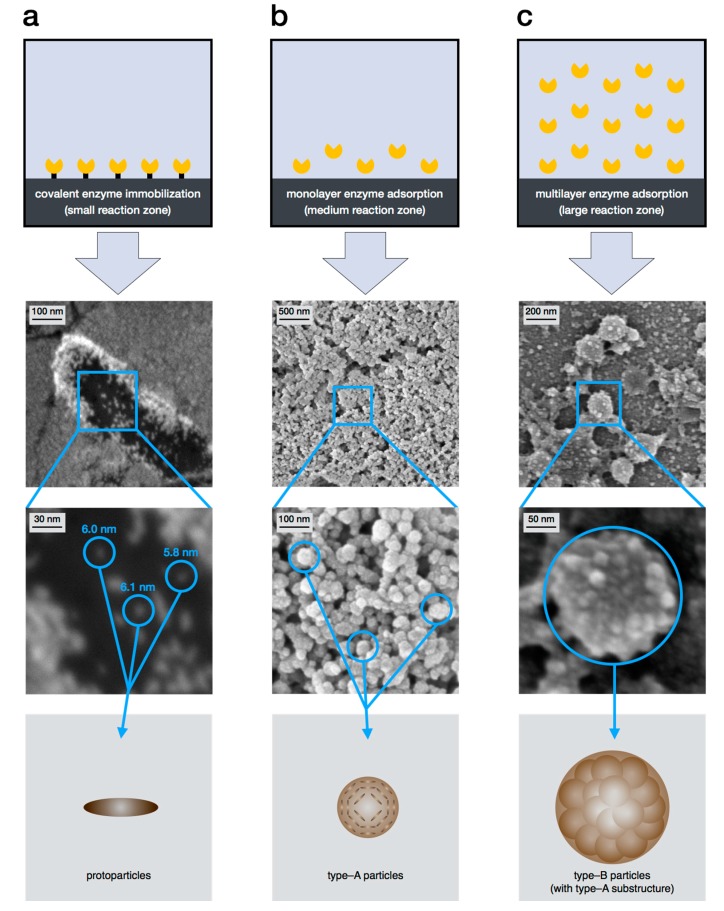
Illustration of the relationship between the reaction zone, adjusted by the choice of immobilization technique, and the deposited eumelanin particle species. SEM picture with magnification and model picture of (**a**) small reaction zone yields protoparticles; (**b**) medium reaction zone yields type-A particles; (**c**) large reaction zone yields type-B particles [31,42,43].

Utilizing this in situ synthesis of eumelanin particles enabled the first controlled generation of all three particle types in a stable and isolated form [31,42,43]. It could be shown that the achieved aggregation state depends on enzyme mobility, which is adjusted by variation of immobilization methods. The relationship between the deposited eumelanin particle species and the reaction zone is shown in Figure 6. The more rigid the immobilization is, the less the enzyme can move from the support. This directly correlates with the reaction zone in the process. The result of a very low reaction zone by covalent immobilization is the production of protoparticles (Figure 6a), whereas a mediocre reaction zone via monolayer adsorption, i.e., a low amount of enzyme, yields type-A particles (Figure 6b). The formation of type-B particles (Figure 6c), which are also found in bulk reactions and most natural eumelanins, required an even larger reaction zone. This was realized via multilayer adsorption of enzyme and the respective use of a higher amount of tyrosinase.

Enzyme mediated autodeposition has therefore shown the potential to stop the supramolecular buildup at each particle level, and enables isolation of the respective eumelanin particles at will. The limitation of this method is the fact that all particles are deposited on a support. Until now, re-dispersing into free particles has not been investigated. If, however, deposition onto surfaces is the goal anyway, the approach enables not only the control over particle morphology. Moreover, the particle deposition occurs site-specific on enzyme functionalized areas only. In this manner, it is possible to address monolayers, multilayers, or even single nanoparticles in specific patterns onto the support.

## 5. Résumé and Future Perspectives

The increasing commitment in the research on the supramolecular structure of eumelanin has already greatly enhanced the knowledge about this fascinating biopolymer. It has become a lot clearer that at least four levels, of which three show particle morphologies, define the hierarchical structure. Of the three buildup steps, the final one is now understood in good detail, although some questions remain.

Nevertheless, several aspects remain diffuse or even totally in the dark. Especially, the mechanisms and kinetics of the first two buildup steps and the possible influence of enzymatic activity on resulting structures need to be clarified. Also, the control over synthesized particle morphologies will certainly be a focus in future research activities. Here, reverting or inhibition of aggregation are promising approaches. With the method of enzyme mediated autodeposition, it is already possible to control the supramolecular buildup to generate distinct intermediates by changing the reaction zone. Based on these results, it would be a great accomplishment to create dispersions of these isolated intermediates which are still stable and don’t follow the usual aggregation.

The generation of stable dispersions of different particle types could possibly open up versatile applications in material science. Due to the biocompatibility of eumelanin, it could be used for drug delivery or as a novel material for implantology. Furthermore, it could be used as a coating for UV protection, corrosion protection, or light harvesting, and in nanotechnology.
